# Immobilization of trypsin in organic and aqueous media for enzymatic peptide synthesis and hydrolysis reactions

**DOI:** 10.1186/s12896-015-0196-y

**Published:** 2015-08-19

**Authors:** Julia Stolarow, Manuel Heinzelmann, Wladimir Yeremchuk, Christoph Syldatk, Rudolf Hausmann

**Affiliations:** Institute of Process Engineering in Life Sciences, Section II: Technical Biology, Karlsruhe Institute of Technology (KIT), Engler-Bunte-Ring 1, 76131 Karlsruhe, Germany; Institute of Food Science and Biotechnology, Section Bioprocess Engineering (150k), University of Hohenheim, Garbenstr. 25, 70599 Stuttgart, Germany

## Abstract

**Background:**

Immobilization of enzymes onto different carriers increases enzyme’s stability and reusability within biotechnological and pharmaceutical applications. However, some immobilization techniques are associated with loss of enzymatic specificity and/or activity. Possible reasons for this loss are mass transport limitations or structural changes. For this reason an immobilization method must be selected depending on immobilisate’s demands. In this work different immobilization media were compared towards the synthetic and hydrolytic activities of immobilized trypsin as model enzyme on magnetic micro-particles.

**Results:**

Porcine trypsin immobilization was carried out in organic and aqueous media with magnetic microparticles. The immobilization conditions in organic solvent were optimized for a peptide synthesis reaction. The highest carrier activity was achieved at 1 % of water (v/v) in dioxane. The resulting immobilizate could be used over ten cycles with activity retention of 90 % in peptide synthesis reaction in 80 % (v/v) ethanol and in hydrolysis reaction with activity retention of 87 % in buffered aqueous solution. Further, the optimized method was applied in peptide synthesis and hydrolysis reactions in comparison to an aqueous immobilization method varying the protein input. The dioxane immobilization method showed a higher activity coupling yield by factor 2 in peptide synthesis with a maximum activity coupling yield of 19.2 % compared to aqueous immobilization. The hydrolysis activity coupling yield displayed a maximum value of 20.4 % in dioxane immobilization method while the aqueous method achieved a maximum value of 38.5 %. Comparing the specific activity yields of the tested immobilization methods revealed maximum values of 5.2 % and 100 % in peptide synthesis and 33.3 % and 87.5 % in hydrolysis reaction for the dioxane and aqueous method, respectively.

**Conclusions:**

By immobilizing trypsin in dioxane, a beneficial effect on the synthetic trypsin activity resilience compared to aqueous immobilization medium was shown. The results indicate a substantial potential of the micro-aqueous organic protease immobilization method for preservation of enzymatic activity during enzyme coupling step. These results may be of substantial interest for enzymatic peptide synthesis reactions at mild conditions with high selectivity in industrial drug production.

## Background

Enzyme immobilization is a method suitable for enzyme reutilization and stabilization possibly leading to lower process costs [1]. However, the number of different immobilization techniques, as well as enzyme carriers, is diverse and complicating. The choice for the best suitable carrier as well as immobilization method has to be done depending on the immobilizate’s application.

Non-porous magnetic microparticles feature a good superparamagnetic separation characteristics, as well as a relatively small particle size of approximately 1 μm, which makes them less prone to mass transport limitations [2]. Due to the particle size, the specific surface available for enzyme coupling is increased compared to other non-porous supports [3].

Micro-aqueous organic media have beneficial effects on enzymatic stability in synthesis reactions where the enzymes may become more rigid and thus less susceptible to conformational changes in the media [4]. Proteins have been reported to show a “pH-memory” of the last aqueous medium prior to lyophilization and are able to maintain their ionization state in micro-aqueous organic solvents [5, 6]. When lyophilized from a pH corresponding to the optimum pH, the activity of the lyophilized and organic solvent resubstituted enzyme has been described to be at its maximum [6]. These effects have been investigated among different enzymes catalyzing synthesis reactions in organic media [7–9].

Trypsin catalyzes hydrolysis reactions, as well as peptide synthesis reactions, when the equilibrium is pushed on either hydrolysis or synthesis product side [10, 11]. This process of protease usage in peptide synthesis and modification reactions is often assumed to offer a more selective and environmentally-friendly option for peptide-hormones and other oligo-peptide-based bio-active agents compared to chemical solid-phase syntheses [12]. Therefore the usage of immobilized trypsin for these kinds of reactions is desirable in order to lower the costs of the process. The application of organic solvent systems for immobilization of hydrolases is so far limited to few publications. One of the first investigations on immobilization in organic solvent was conducted for lipase [13]. The researchers compared aqueous, microemulsion and organic systems for immobilization to test the lipase activity in a hydrolysis and transesterification reactions. Within transesterification reaction the organic and microemulsion systems showed 20 and 40 % residual activities, respectively, for immobilization while within aqueous method no transesterification activity after immobilization could be detected. In subsequent works lipase from *Candida rugosa* was immobilized in apolar and polar solvents such as hexane and acetone and compared to aqueous immobilization in buffer [14, 15]. The results showed a sevenfold higher specific activity of acetone immobilized lipase in butyl butyrate synthesis reaction compared to aqueous solution immobilization. Sun et al. [16] immobilized lipase from *Candida antarctica* by adsorption in isooctane. This immobilization achieved an up to threefold higher activity recovery compared to adsorption in aqueous medium. Zhu et al. [17] immobilized several enzymes including trypsin within micro-aqueous organic media onto chitosan microspheres. Immobilization under micro-aqueous conditions showed a twofold higher remaining catalytic activity compared to water phase immobilization, which appears to be a promising alternative to the mostly used aqueous immobilizations.

In the present study an immobilization method in organic solvent was analyzed considering different immobilization parameters influencing the protein coupling and activity coupling. The enzymatic activity was measured in a dipeptide synthesis reaction. The operational stability was determined in ten sequential peptide synthesis and hydrolysis cycles. Furthermore, the elaborated immobilization method was compared to an aqueous media immobilized trypsin by such factors as protein coupling yield, activity coupling yield, specific activity yield. A special focus was set on the comparison of these immobilization methods in both possible trypsin-catalyzed reaction types: the hydrolysis reaction which is performed at physiological conditions in aqueous medium and synthesis reaction in organic medium which plays an important role in industrial drug production.

## Results and discussion

### Enzymatic synthesis of Bz-Arg-Arg-NH_2_

**Scheme 1:**
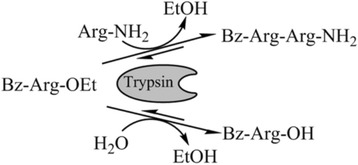
Trypsin-catalyzed conversion of Bz-Arg-OEt to a dipeptide amide and a competing hydrolysis reactions

For the synthesis of Bz-Arg-Arg-NH_2_, which may act as an intermediate of several bioactive peptides, a kinetically controlled synthesis approach was selected [18, 19]. The reaction overview is given below. Bz-Arg-OEt was used as C-terminus activated and N-protected substrate. Trypsin binds Bz-Arg-OEt under release of ethanol forming an acyl intermediate which can be attacked by a nucleophile such as an amino containing component or water. In this reaction Arg-NH_2_ acts as a nucleophile forming a peptide bond where the dipeptide Bz-Arg-Arg-NH_2_ is released (see reaction Scheme 1). The optimum molar ratio of Bz-Arg-OEt to Arg-NH_2_ amounts to a molar excess of the nucleophile of 1:20. Formation of dipeptide in this reaction was confirmed by MALDI-ToF-MS analysis with an expected molecular mass of [M + H] + = 434.26 Da and a found molecular mass of 433.73 +/− 0.5 Da.

### Screening for suitable solvent and parameter optimization for enzyme immobilization

For optimization of trypsin immobilization in organic medium a screening for the best suitable organic solvent was accomplished. A constant protein input of 0.025 g_trypsin_/g_particle_ was used. The results showed dioxane as the best suitable medium for trypsin carrier activity upon immobilization (Table 1). However, no correlation to the solvent properties was found. For further optimization, three influence factors for immobilization method were tested: concentration of the activating agent CDI, activating and coupling time. The experiments showed a minor influence of CDI in the tested range of 0.12-2.5 g_CDI_/g_particle_. The lowest tested amount of CDI of 0.12 g_CDI_/g_particle_ results in 740 μmol_CDI_/g_particle_ and may activate approximately 78 % of the functional COOH-groups on magnetic particle surface. However, the functional group density of a magnetic particle exceeds the available particle surface for trypsin coupling, so that a smaller amount of activated COOH-groups is sufficient. The increasing of activating time in a range between 6–149 min had a positive effect on carrier activity and specific activity yield. And finally an increase in coupling time in the tested range of 1–6 h achieved an increasing carrier activity and specific activity yield, but decreasing protein coupling yield and activity coupling yield. The final optimum conditions for trypsin immobilization in dioxane were 0.12 g_CDI_/g_particle_, 82 min activating time and 1 h coupling time.

**Table 1:** Relative carrier activity for trypsin immobilization in different organic solvents with various physical properties

	Relative carrier activity/%	Log K_OW_^a^	Relative polarity^b^
Methanol	-	−0.74	0.762
Ethanol	-	−0.3	0.654
Acetone	45.1	−0.24	0.355
1,4-Dioxane	100.0	−0.27	0.164
1-Propanol	81.2	0.25	0.617

-no activity could be detected within this method

^a^log K_OW_ values were extracted from GESTIS data base of chemicals

^b^rel. polarity_water_ = 1, values from Reichardt [20]

### Influence of water content in dioxane on protein coupling and carrier activity during immobilization

**Fig. 1:**
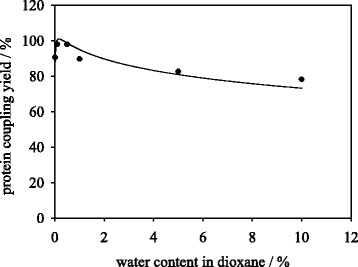
Protein coupling yield of trypsin immobilization onto M-PVA in dioxane containing variable water content. Data points were fitted using log normal three parameter equation

As a significant factor for trypsin immobilization the water content (v/v) in dioxane during enzyme coupling was elaborated. Trypsin was used in lyophilized form in order to control the water content in dioxane and conserve the enzymatic ionization state from the last aqueous solution during protein coupling step. Trypsin was used in 0.025 g_trypsin_/g_particle_ ratio. The trypsin suspension in different dioxane/water solutions exhibited an opaque appearance indicating the enzyme is not fully soluble in dioxane. After particle introduction and coupling step the enzyme suspension in dioxane appeared clear. Higher water contents lead to faster hydrolysis of CDI-activated carboxylic acids, which has a negative effect on the protein coupling yield (Fig. 1). Furthermore, the water content influences the solubility and the conformation of the enzyme during the coupling step. Higher water contents may lead to water saturation of enzyme crystals in organic solvent making the enzymes’ conformation more similar to a fully aqueous environment [21]. By that, the ionization state from lyophilization is altered when organic solvent contains a higher amount of water. Furthermore, as the magnetic particles feature a polar surface the presence of enzyme in dioxane with high water content may prevent the adsorption step of the enzyme to the particles surface required for subsequent covalent binding.

**Fig. 2:**
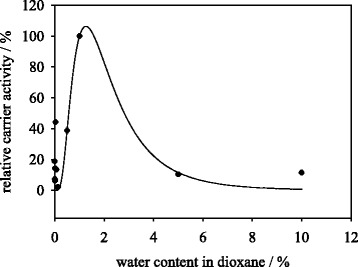
Relative carrier activity of trypsin covalently immobilized onto M-PVA in dioxane containing variable water content. Data points were fitted using log normal three parameter equation

Figure 2 shows the correlation between carrier activity and water content in dioxane. In the work of Zhu et al. [17] a water content of 0.4 % in dioxane was shown to be optimal for trypsin immobilization onto chitosan microspheres. For immobilization of *C. antarctica* lipase in isooctane an optimum water content of 1.4 % (v/v) for residual activity was found by Sun et al. [16]. In our data the data regression shows an optimum protein coupling yield between 0.1 and 0.5 % of water in dioxane, while the highest carrier activity was found at 1.2 % of water in dioxane. For further experiments a water content of 1 % was chosen for best coupling and activity yield during enzyme coupling step.

### Reusability of trypsin immobilized in dioxane

**Fig. 3:**
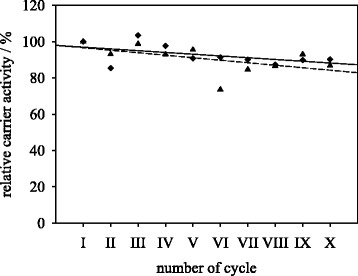
Reusability of trypsin immobilized onto M-PVA in organic solvent in synthesis reaction (♦) and hydrolysis reaction (▲). One cycle of synthesis reaction corresponds to one hour of peptide synthesis reaction in 80 % ethanol at 20 °C, one cycle of hydrolysis reaction corresponds to 30 min in Tris buffered solution pH 8 at 30 °C. Linear trend lines show possible end values of activity for synthesis (—) and hydrolysis (− − −) reactions

In order to determine the stability of organic medium immobilized trypsin in repeated peptide synthesis and hydrolysis reactions, immobilizates produced at optimum conditions in dioxane with a 0.02 g_trypsin_/g_particle_ protein loading were used. Peptide synthesis reaction results are shown in Fig. 3. After ten reuse cycles, the relative carrier activity in peptide synthesis reaction remains at 90 % of initial activity. The hydrolysis activity remains at 87 % after ten cycles. These results show higher operational stability compared to earlier works. Covalent immobilized trypsin in organic solvent retained 68 % of its hydrolytic activity after seven cycles [17]. Porcine pancreatic lipase immobilized covalently in organic media retained 63 % of initial activity after five runs in butyl octanoate synthesis [22]. Earlier works reported on the reusability of enzymes immobilized in organic media by adsorption. De Castro et al. (1999) stated a remaining activity of 40 % after three repeated batches of butanol conversion by porcine pancreatic lipase [14]. In the work of Sun et al. (2010) a synthetic activity of 57 % remained after seven repeated conversions with immobilized lipase from *C. antarctica,* but only 25 % of hydrolytic activity remained after seven cycles [16]. Possible reasons for activity decrease during repeated application of immobilized enzymes are enzyme desorption from the carrier or activity loss due to enzyme inactivation in reaction media. Apparently, covalent binding of enzymes in organic medium circumvents possible desorption of enzyme from the carrier in other organic or aqueous media. In addition, the activity of the immobilized enzyme remains more stable compared to the initial value.

### Comparison of dioxane and aqueous immobilization methods for peptide hydrolysis and synthesis

**Fig. 4:**
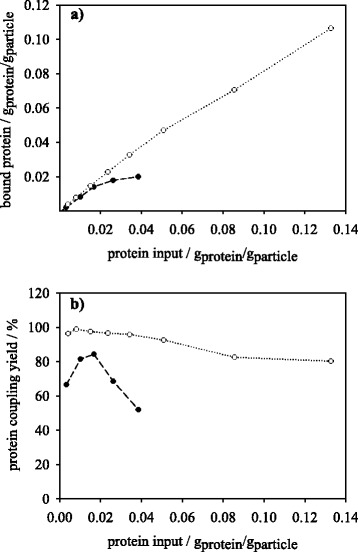
**a** Bound protein in g_protein_/g_particle_ and **b** protein coupling yield as function of protein input for immobilization of trypsin onto M-PVA in dioxane (●) and MES buffer (○)

Since the immobilization of trypsin in organic media has been rarely reported, a comparison to a more common aqueous covalent immobilization technique on magnetic micro-particles is needed in order to assess the elaborated method in a peptide synthesis and peptide hydrolysis reactions. The protein input for each immobilization method was varied between 0.001 and 0.133 g_protein_/g_particle_. Immobilization in organic solution was conducted at 1 % (v/v) water in dioxane at optimum conditions. For a comprehensive assessment of the two techniques response factors such as protein coupling yield, specific activity yield and activity coupling yield were calculated for peptide synthesis as well as peptide hydrolysis assays and plotted against protein loading in g_protein_/g_particle_. In Fig. 4a the binding of protein over protein input is shown. Within aqueous immobilization method the binding increases almost linearly with protein input. By dioxane immobilization a similar progress of protein binding with protein input up to a value of 0.02 g_protein_/g_particle_ is shown. With protein inputs above this value the protein binding reveals saturation where the amount of bound protein does not exceed 0.02 g_protein_/g_particle_. Figure 4b shows the results of protein coupling yield for both tested methods. Within aqueous method protein loadings of up to 0.04 g_protein_/g_particle_ with 95 % and higher protein coupling yields are possible and with protein inputs higher than 0.04 g_protein_/g_particle_ the protein coupling yield diminishes to 80 %. For the dioxane immobilization method the protein coupling yield values are significantly lower. Highest protein coupling yields of 80–84 % are reached at 0.01–0.02 g_protein_/g_particle_. The amount of bound trypsin molecules in dioxane is limited by the distribution and solubility of trypsin in this solvent. This result is confirmed by the work of Malmsten and Larsson [23]. Trypsin was immobilized in a water-in-oil microemulsion. The stability of trypsin in this emulsion was limited by the maximum protein concentration that could be applied in the stated method.

**Fig. 5:**
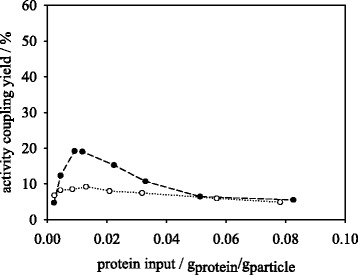
Effect of protein input on peptide synthesis activity coupling yield of trypsin immobilized in dioxane (●) and MES buffer (○)

**Fig. 6:**
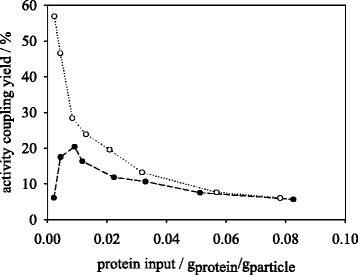
Effect of protein input on peptide hydrolysis activity coupling yield of trypsin immobilized in dioxane (●) and MES buffer (○)

The synthesis and hydrolysis activity coupling yields for dioxane and aqueous immobilization methods are depicted in Figs. 5 and 6. Within synthesis reaction (Fig. 5) the activity yields of different protein loadings by aqueous method behave almost constant at values of 6–8 %. However, the dioxane method may reach activity coupling yields of up to 20 % showing higher values than the aqueous method within a protein input range of 0.004–0.04 g_protein_/g_particle_. The activity coupling yield for the dioxane method experiences a drop with protein input of >0.012 g_protein_/g_particle_. One reason for this behavior is the decreasing protein loading yield in dioxane immobilization which leads to constant protein loadings above 0.02 g_protein_/g_particle_ protein input (Fig. 4a). This immobilization method presents a similar behavior in the hydrolysis reaction to the synthesis reaction (Fig. 6). The maximum activity coupling yield amounts to 20 %. For the aqueous immobilization method a decrease in activity coupling yield with increasing protein input from 57 to 5.6 % in hydrolysis (Fig. 6) was observed. Since the protein coupling yield in aqueous method amounts to 95–100 % up to a protein input of 0.04 g_trypsin_/g_particle_ the decrease in activity coupling indicates a limitation of substrate transport in the medium. Especially, as the introduced substrate concentration of 1 mM BAPNA in this reaction is below the stated *K*_*m*_ value of trypsin for BAPNA of 1.5 mM [24].

**Fig. 7:**
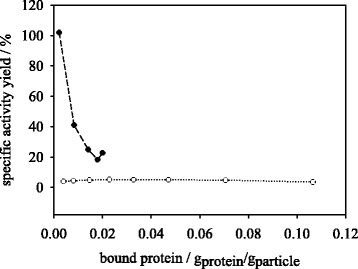
Effect of protein input on specific peptide synthesis activity yield of trypsin immobilized in dioxane (●) and MES buffer (○)

**Fig. 8:**
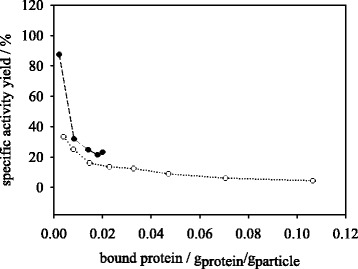
Effect of protein input on specific peptide hydrolysis activity yield of trypsin immobilized in dioxane (●) and MES buffer (○)

Considering the specific activity yields as a function of actual bound protein in g_trypsin_/g_particle_ the two methods show a diverging behavior in the peptide synthesis reaction. The synthetic activity is depicted dependent on bound protein in Fig. 7. The results of aqueous immobilization indicate a rapid decrease of specific activity within very low protein concentrations of <0.004 g_protein_/g_particle_ reaching a constant value of 4–5 % above this protein loading. Within dioxane immobilization method the specific activity yield starts with 100 % at 0.002 g_protein_/g_particle_ decreasing to 20 % at 0.02 g_protein_/g_particle_. Considering the specific hydrolysis activities the two methods show stronger convergence (Fig. 8). In the dioxane immobilization method a specific activity drop from 88 % at 0.002 g_protein_/g_particle_ to 23.3 % at 0.02 g_protein_/g_particle_ occurs, while in the aqueous method the specific activity starts at 33.3 % for 0.004 g_protein_/g_particle_ and decreases to a value of 4.4 % at 0.11 g_protein_/g_particle_. As mentioned above, the main reason for the decrease in specific immobilized trypsin activity within synthesis and hydrolysis reactions may be the limitation of substrate available to the bound enzyme on the particle surface. Within the synthesis reaction the immobilizate is less susceptible to mass transport limitation, as the catalysis reaction is slower than in hydrolysis and higher substrate concentrations (10 mM Bz-Arg-OEt, 1.5 U/mg_free trypsin_) are used. Compared to the synthesis reaction, the reaction rate in hydrolysis is higher and the concentration of substrate is lower (1 mM BAPNA, 2.7 U/mg_free trypsin_). Substrates with higher molecular weights may influence the mass transport to the immobilized enzyme, but not alter the observed difference in immobilization methods since it is based on conformational effects. Furthermore, it is conceivable, that the synthesis reaction is more substrate specific and dependent of a certain enzyme conformation state. The BAPNA hydrolysis reaction may occur at slow, basic levels even without introduction of a biocatalyst and may hence require a less conserved enzyme conformation. Since organic immobilization may preserve a more active conformation of trypsin compared to the aqueous method, this effect may be more prominent in the synthesis reaction. Consequently, the results show that the use of organic method for immobilization of trypsin at protein loading of 0.01 g_protein_/g_particle_ and lower may improve the conversion of substrates in synthesis and hydrolysis reactions, compared to an aqueous covalent binding method. These findings are supported by observations made by Stark and Holmberg [13], who immobilized lipase from *Rhizopus sp*. in aqueous and organic systems in order to perform hydrolysis and transesterification reactions. The authors stated that lipase immobilized under organic conditions was more active in transesterification reaction whereas lipase immobilized in buffer showed no activity at all. In hydrolysis reaction the differences between the compared methods were less pronounced.

## Conclusions

In the present study a method for covalent trypsin immobilization on magnetic particles in organic solvent was investigated. The used magnetic particles were stable within several organic media and suitable for trypsin immobilization. Trypsin immobilized within dioxane solvent conditions showed a higher specific activity and activity coupling yield in peptide synthesis reaction. For peptide hydrolysis the aqueous method achieved better results regarding activity coupling yield over a protein input range of 0.001–0.05 g_protein_/g_particle_, but was similar to dioxane immobilization in specific activity yield. These results indicate a substantial potential of the organic protease immobilization method for preservation of enzymatic activity during enzyme coupling step. The results revealed, that the organic method suffers from enzyme solubility limitations at given conditions. Consequently, future investigations should focus on improvement of protein solubility for immobilization in organic medium and testing of protease immobilization in water-immiscible organic solvents.

## Methods

Magnetic poly(vinyl alcohol) micro-particles (M-PVA) C22–C250 were provided by PerkinElmer Chemagen, Baesweiler, Germany. The used beads have a carboxyl functionalization with a concentration of 950 μmol COOH/g_particle_ and particle size distribution within 1–3 μm. 1,4-dioxane, 1-propanol, methanol, ethanol, acetone, acetonitrile, 2-(N-morpholino)ethanesulfonic acid (MES), carbonyldiimidazol (CDI), calcium chloride, tris(hydroxymethyl)aminomethane (Tris) and trifluoroacetic acid (TFA) were purchased from Carl Roth, Karlsruhe, Germany. N-(3-Dimethylaminopropyl)-N′-ethylcarbodiimide hydrochloride (EDC), Nα-Benzoyl-L-arginine ethyl ester hydrochloride (Bz-Arg-OEt), Nα-Benzoyl-DL-arginine 4-nitroanilide hydrochloride (BAPNA), p-nitroaniline (pNA) and L-arginine amide dihydrochloride (Arg-NH_2_) were purchased from Sigma-Aldrich, Deisenhofen, Germany. Recombinant porcine trypsin (EC 3.4.21.4) was purchased from Roche Diagnostics, Mannheim, Germany. Nα-Benzoyl-arginyl-arginine amide trifluoroacetate (Bz-Arg-Arg-NH_2_) was synthesized by Bachem, Weil am Rhein, Germany. Nα-Benzoyl-L-arginine (Bz-Arg) was obtained from ABCR GmbH, Karlsruhe, Germany. BC Assay was performed by a BC Assay Protein Quantitation Kit from Interchim, Uptima, Montluçon, France. HPLC analysis was performed with a standard HPLC device from Agilent 1200 Series, Agilent, Waldbronn, Germany equipped with a reversed phase column (C18 Luna, 5 μm, 250 × 4.60 mm, Phenomenex, California, USA).

### Covalent immobilization of trypsin in organic and aqueous media

For immobilization in organic media the protocol was as follows. The M-PVA particle suspension concentration was adjusted to 25 mg/ml and washed with 0.1 M MES buffer pH 5.3 for three times followed by four washes in dried 1,4-dioxane with >99.8 % purity. For activation of functional COOH-groups on particle surface CDI was used as activating substance at 0.12 g_CDI_/g_particle_ and incubated with particles at 18 °C for 82 min. After activation particles were washed with 1.4-dioxane for two times. Trypsin was lyophilized at pH 7 prior to immobilization, suspended in dioxane with given water content from 0 to 10 % at concentrations of 0.002–0.080 mg/g_particle_ and incubated at room temperature for 1 h.

For immobilization in aqueous media a modified protocol according to Hermanson (2008) [25] was applied. The pH 5.3 adjusted 25 mg/ml particle suspension was activated by a 1.6 g_EDC_/g_particle_ EDC solution for 6 min at 11 °C and washed two times with 0.1 M MES buffer pH 7. Trypsin solutions in a range of 0.002–0.080 g/g_particle_ in 0.1 M MES pH 7 were added to the particles and incubated for 30 min at 25 °C for enzyme coupling.

After enzyme coupling step the particles from each immobilization method were washed with 0.04 M Tris buffer pH 9 for three times and stored in 0.02 M CaCl_2_ solution at 4 °C. The coupling supernatant and washing solutions were used for quantification of the bound enzyme by BC Assay. The particle concentration after immobilization was determined gravimetrically by drying 250 μl of the respective immobilizate at 70 °C for at least 16 h in micro-tubes.

### Synthetic and hydrolytic activity measurement of free and immobilized trypsin

For measurement of synthetic trypsin activity 200 μl of a 25 mg/ml immobilizate suspension were used removing the supernatant by magnetization. 1.5 ml of a substrate solution containing 10 mM Bz-Arg-OEt, 200 mM Arg-NH_2_ in 80 % (v/v) ethanol and 20 % 0.1 M Tris buffer pH 9 with 0.02 M CaCl_2_ was added to the immobilizates starting the synthesis reaction. Samples were taken without particle uptake and mixed in a 1 to 4-ratio with 6 % (v/v) TFA in order to terminate the reaction. For free trypsin an enzyme concentration of 0.1 mg/ml in 1.5 ml substrate solution was used. Samples were analyzed by HPLC. 1 Unit is defined as 1 μmol of synthesized Bz-Arg-Arg-NH_2_/min of reaction.

Hydrolytic activity determination was accomplished using a 1 mM BAPNA solution in 0.1 M Tris buffer pH 8 containing 0.02 M CaCl_2_. The reaction was conducted at 30 °C and initiated by introduction of 1.5 ml of substrate solution to 200 μl particles without supernatant. Samples were taken and the reaction was terminated by a 1 to 4-ratio of sample to 6 % TFA solution. For determination of free enzyme activity a solution of 0.01 mg_trypsin_/ml in 1.5 ml of substrate was used. Samples as well as pNA standard were analyzed in a microplate reader at 405 nm. 1 Unit is defined as 1 μmol of pNA released per minute of hydrolysis reaction.

### Protein quantification assay

Protein quantification of protein stock solutions and supernatants was performed using a standard BC Assay Protein Quantitation Kit. The assay was used in order to quantify the protein loading of immobilized enzyme measured against bovine serum albumin standard in the respective buffer solution.

Response parameters (Equations 1, 2, 3, 4 and 5) could be calculated after product quantitation in each individual assay for different immobilization methods.

1 $$ \mathrm{protein}\ \mathrm{coupling}\ \mathrm{yield}=100\%\times \frac{\mathrm{experimental}\ \mathrm{protein}\ \mathrm{loading}/{\mathrm{g}}_{\mathrm{bound}\ \mathrm{protein}}/{\mathrm{g}}_{\mathrm{particle}}}{\mathrm{theoretical}\ \mathrm{maximum}\ \mathrm{protein}\ \mathrm{loading}/{\mathrm{g}}_{\mathrm{protein}\ \mathrm{input}}/{\mathrm{g}}_{\mathrm{particle}}}. $$

2 $$ \mathrm{specific}\ \mathrm{carrier}\ \mathrm{activity}=\frac{\mathrm{U}}{{\mathrm{g}}_{\mathrm{particle}}}. $$

3 $$ \mathrm{activity}\ \mathrm{coupling}\ \mathrm{yield}=100\%\times \frac{\mathrm{experimental}\ \mathrm{specific}\ \mathrm{carrier}\ \mathrm{activity}/\mathrm{U}}{\mathrm{theoretical}\ \mathrm{specific}\ \mathrm{carrier}\ \mathrm{activity}\ \mathrm{free}\ \mathrm{enzyme}\kern0.5em /\mathrm{U}}. $$

4 $$ \mathrm{specific}\ \mathrm{activity}=\frac{\mathrm{U}}{{\mathrm{g}}_{\mathrm{protein}}}. $$

5 $$ \mathrm{specific}\ \mathrm{activity}\ \mathrm{yield}=100\%\times \frac{\mathrm{experimental}\ \mathrm{specific}\ \mathrm{activity}/\mathrm{U}/{\mathrm{g}}_{\mathrm{bound}\kern0.5em \mathrm{protein}}}{\mathrm{specific}\ \mathrm{activity}\ \mathrm{free}\ \mathrm{enzyme}\ /\mathrm{U}/{\mathrm{g}}_{\mathrm{bound}\kern0.5em \mathrm{protein}}}. $$

### High pressure liquid chromatography (HPLC) analysis of peptide synthesis samples

Peptide synthesis samples were analyzed by RP-HPLC-UV/Vis in a 18 %/82 % (v/v) ACN/H_2_O solution as mobile phase containing 0.05 % TFA, at a column temperature of 60 °C for 13 min at 254 nm. As standard substances Bz-Arg-OEt, Bz-Arg, and Bz-Arg-Arg-NH_2_ were used in order to quantify the product concentrations.
